# What influences newly graduated registered nurses’ intention to leave the nursing profession? An integrative review

**DOI:** 10.1186/s12912-023-01685-z

**Published:** 2024-01-19

**Authors:** Xiao-Chen Lyu, Shuang-Shuang Huang, Xiao-Ming Ye, Lu-Yu Zhang, Peng Zhang, Ya-Juan Wang

**Affiliations:** 1 The Outpatient Operating Room, the First Affiliated Hospital of Wannan Medical College, Wuhu, China; 2 Neurology Intensive Care Unit, the First Affiliated Hospital of Wannan Medical College, Wuhu, China; 3 Intensive Care Unit, the First Affiliated Hospital of Wannan Medical College, Wuhu, China; 4 School of Nursing, Wannan Medical College, Wuhu, China

**Keywords:** Intention to leave, Newly graduated registered nurses, Novice registered nurses, Integrative review, Young registered nurses

## Abstract

**Background:**

Newly graduated registered nurses leaving the nursing profession in the early stages of their career have enormous financial and time implications for nursing organizations and affect the quality of nursing care.

**Objective:**

To identify the factors influencing newly graduated registered nurses’ intention to leave the nursing profession over the past 10 years.

**Methods:**

The framework developed by Whittemore and Knafl was used to conduct this integrative review. An electronic search was conducted for English articles to identify research studies published between 2011-2022 using the following databases of PubMed, MEDLINE, CINAHL, PsycINFO, and Scopus. Eligible publications were critically reviewed and scored using the Critical Appraisal Skills Program Checklist and the Center for Evidence-Based Management appraisal.

**Results:**

Twenty-one studies were analyzed. The main factors affecting newly graduated registered nurses’ intention to leave the nursing profession included demographic factors (age, educational level, year of experience, professional title, employment status, health status, shift, hospital location and size), supervisor and peer support, challenges in the workplace, cognitive and affective response to work, work environment (collegial nurse-physician relations, insufficient staffing level, person-work environment fit), gender stereotypes, autonomous motivation, role models, and resilience.

**Conclusions:**

The factors affecting newly graduated registered nurses’ intention to leave the nursing profession are multifaceted and should receive continuous attention from nurse managers. The findings provide more comprehensive for nurse administrators to develop intervention strategies to mitigate newly graduated registered nurses’ turnover intention.

## Introduction

Nurses are important members of healthcare systems, providing quality nursing care and ensuring patient safety. Newly graduated registered nurses (NGRNs) was defined by Benner [1] as not having any previous clinical experience regarding patient management and responsibility; and being unfamiliar with hospital policies, procedures, protocols, and tools. NGRNs need time to integrate skills and practice in the workplace to become experienced registered nurses (RNs). However, previous study reported that the transition from NGRNs to the role of experienced RNs become more difficult [2]. It takes them at least one year to feel comfortable and confident practicing in the clinical setting. Meanwhile, the process of nurse attrition from the nursing workplace begins after graduation and persists throughout the nurse’s career [3]. Compared to the experienced RNs, NGRNs lack the necessary clinical skills at the primary stage of their career [4]; heavy workload and high job stress in their first year of practice will contribute to decreased job satisfaction and hence NGRNs desire to leave the nursing profession [5].

The attrition rate of NGRNs remains at a high level worldwide. This is worrisome given the large number of resources invested in NGRNs. The Joint Commission report, undertaken in the United Kingdom reported that one in four NGRNs plan to leave their first posts within the first 12 months after registration. Sandler reported that in Canada, 18% to 30% of NGRNs choose to leave the nursing profession in their first year, and from 37% to 57% left in their second year [6]. In Turkey, 42.5% of NGRNs considered leaving the nursing profession in their first year [7]. Such nursing turnover aggravates the existing nursing shortage, directly impacting the quality of nursing care and patient safety [8]. Meanwhile, high nurse turnover can lead to increased healthcare costs and adverse patient outcomes [9]. Through the literature review, we found that various factors influence nurses’ intention to leave their organization including demographics, quality of work life, job satisfaction, leadership style, organizational commitment, work environment, job satisfaction, motivation, job security, family reason, and bullying at work [10]. However, there is a vast difference in terms of intention to leave (ITL) nursing profession between NGRNs and experienced RNs. Therefore, it is necessary to understand the influencing factors of NGRNs’ ITL, so that specific strategies targeting NGRNs retention can be further developed and implemented.

This integrative review aims to identify influencing factors on NGRNs’ ITL the nursing profession and demonstrate gaps in the existing knowledge base. A preliminary search of PubMed, MEDLINE, CINAHL, PsycINFO, and Scopus was conducted, with no published or ongoing systematic reviews on NGRNs’ ITL the nursing profession identified. ITL and intention to stay (ITS) are two sides of the same coin; thus, in this review, the terms ITL, turnover intention, and ITS are used interchangeably. Additionally, the results of this integrative review may help administrators and health policymakers of the various healthcare services provided by hospitals and home healthcare agencies focus on organizational efforts and retention strategies that can help reduce NGRNs turnover.

## Methods

The framework developed by Whittemore and Knafl [11] was used to conduct this integrative review of the literature, which consists of five stages: problem identification, literature search, data evaluation, data synthesis, and presentation of findings. This approach allows for the identification and synthesis of evidence sourced from studies that used diverse methodologies (for example quantitative study, qualitative survey, and mixed method) and hence gain a holistic understanding of this topic.

### Literature search

Based on the Whittemore and Knafl methodology [11], the first stage is problem identification, the following question was set to answer the study’s aim: What influences newly graduated registered nurses’ intention to leave the nursing profession? In the second stage (literature search), an electronic search was conducted for English articles to identify research studies published between 2011-2022 using the following database of PubMed, MEDLINE, CINAHL, PsycINFO, and Scopus. The above databases cover almost all published articles related to this review topic and the characteristics of the recorded publications in these databases are high-impact factors, peer-reviewed journals, top-ranked journals by industry studies, and good quality. These databases can complement each other and help us obtain more comprehensive literature. The following two groups of search terms were used in combination: (a) newly graduated registered nurses, novice registered nurses, and young registered nurses; (b) intention to leave, turnover intention, intention to quit, and intention to stay. In this stage, the results of the comprehensive search included 441 articles after reviewing them based on the inclusion criteria such as: accessing the full text of the article, including the keywords in the title and abstract of the article, and writing in English, finally 420 were removed, and the 21 articles were included.

### Eligibility criteria

Included articles were peer-reviewed studies, published in English, which investigated NGRNs’ ITL the nursing profession and related influencing factors. Articles were limited to those published between 2011 and 2022 to reflect the most recent evidence within the last decade.

### Study selection and data extraction

In this stage (data evaluation), after excluding duplicate articles, two authors independently reviewed the titles, abstracts, and full texts following the Critical Appraisal Skills Program (CASP) Checklist [12] and the Center for Evidence-Based Management (CEBM) appraisal [13]. When they disagreed on an article, a third author joined the discussion until a consensus was reached. The Preferred Reporting Items for Systematic Reviews and Meta-Analysis (PRISMA) diagram outlines the screening process used in literature searches.

### Data synthesis

In the fourth stage (data synthesis), the researchers followed the data synthesis approach to analyze included studies. There were four steps: (a) Data reduction. The included articles were divided into subgroups (quantitative surveys, qualitative research, and mixed studies) for ease of analysis. (b) Data display. Open coding of each included study was performed by three authors. After many iterations and discussions related to this coding process, the three authors reached a consensus on the formulation of themes and sub-themes of the influencing factors of NGRNs’ ITL the nursing profession. (c) Data comparison. This involved an iterative process of examining data displays of primary source data in order to identify patterns, themes, or relationships. (d) Drawing conclusions and verification. This moved the interpretive effort from the description of patterns and relationships to higher levels of abstraction, subsuming the particulars into the general.

Finally, confirmation and verification were conducted by all researchers to ensure that all twenty-one articles were thoroughly assessed at all methodological stages and that the results matched the research questions of the study.

### Quality appraisal

Whittemore and Knafl [11] pointed out that evaluating the quality of the evidence included is not essential in supplementary reviews. All articles meeting the inclusion criteria, regardless of their methodological quality, were retained in this review to examine all evidence on factors influencing the implementation of the nursing role in practice settings. In addition, the Critical Appraisal Skills Program (CASP) Checklist [12] and the Center for Evidence-Based Management (CEBM) appraisal [13] were used to assess the studies, which allowed us to evaluate and compare research objectives, design, methods, analysis, results, discussion, conclusions, and implications. In this integrative review, studies were considered relatively high quality, while studies in the moderate or low-quality range were omitted.

### Ethics

This study was conducted based on published data and ethical approval was not required.

## Results

According to the framework of Whittemore and Knafl [11], the last step is the presentation of findings. The results of twenty-one included studies in this review were presented below:

### Search results

**Fig. 1:**
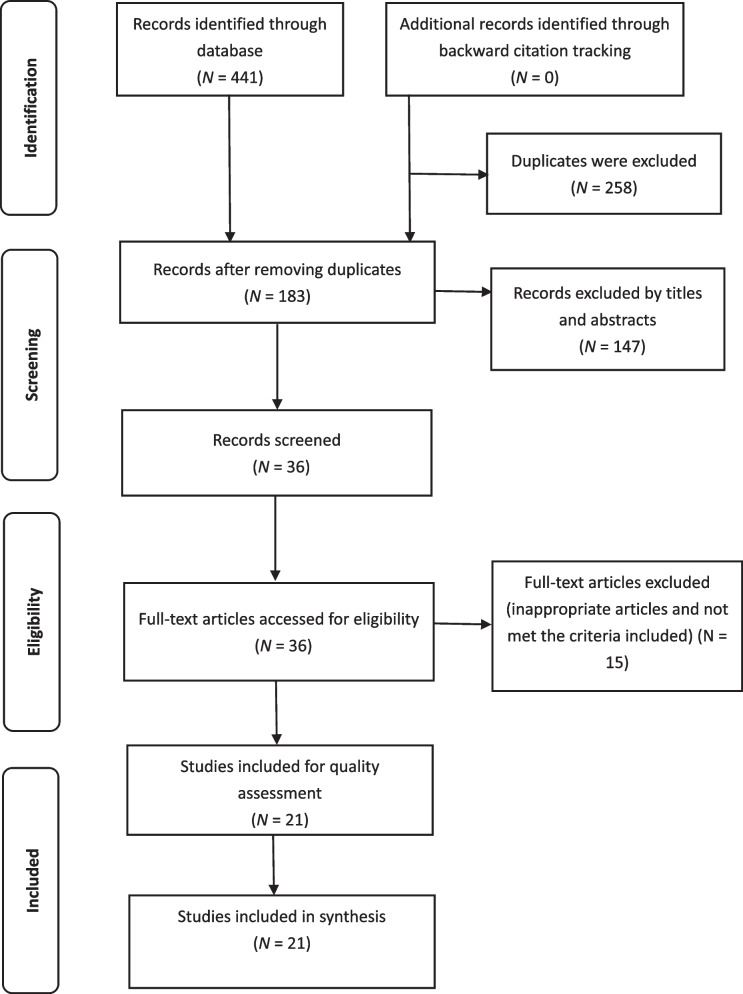
PRISMA flow diagram

Four hundred and forty-one titles and abstracts were identified from five databases. In total, two hundred and fifty-eight articles were excluded due to duplicates and one hundred eighty-three articles were screened by two authors independently by title and abstract. After excluding one hundred forty-seven articles by titles and abstracts, thirty-six articles were reviewed in full text. Finally, fifteen articles were inappropriate and did not meet the inclusion criteria and were therefore excluded. Overall, twenty-one articles were included, two qualitative studies and nineteen quantitative surveys; no mixed methods studies were included (Figure 1).

### Study characteristics

Twenty-one studies were included in the final review, and the main characteristics of the included studies were extracted (Table 1). The included studies were conducted in eight countries, five studies in the United States, four studies in Canada, three studies in Mainland of China, two studies in Japan, two studies in Taiwan, and one study each in Turkey and Australia.

**Table 1:** Bibliography of included studies

**No.**	**Reference**	**Country**	**Sample**	**Research type and method**	**Instruments**	**Main results/findings**
1	Tominaga & Miki (2011) [14]	Japan	1364 newly graduated nurses from 14 university hospitals	Quantitative research A cross-sectional study	A six-item of Intention to Leave Scale; A 21-item Job Readiness Scale; The Japanese short version of the Effort-Reward Imbalance Questionnaire (ERI); Four-item Role model scale; 12-item General Health Questionnaire (GHQ-12)	Japanese newly graduated nurses have a low level of intention to leave the nursing profession. This study revealed that effort, subjective health status, role models, and effort were important factors that related to the newly graduated nurses’ intention to leave, as well as their age and city size. Over-commitment and reward were not significant factors.
2	Peterson et al., 2011 [15]	Canada	232 newly graduated nurses working in acute care hospitals	Quantitative research A cross-sectional survey	A seven-item of Workload Scale; 20 items of Job Control Scale; the Social Support Scale; the Personal Efficacy Beliefs Scale; the Job Satisfaction Scale	Job demands were significantly and positively related to intention to leave the job. However, support from coworkers negatively were related to intention to leave the job. It was also found that new nurses working at a teaching hospital were more likely to report lower intention to leave the job than those working in small or community hospitals.
3	Brewer et al., 2012 [16]	USA	1653 newly licensed registered nurses from the whole USA	Quantitative research Longitudinal panel design of two survey 1 year apart	The Intention to Stay Scale; the Search Behavior Scale; the Organizational Commitment Scale; the Supervisory Support Scale; the Mentor Support Scale; the Work Group Cohesion Scale; the Autonomy and Variety Scale; the Distributive Justice Scale; the Procedural Justice Scale; the Work-family Conflict Scale; the Promotional Opportunities Scale; the Collegial RN-MD Relations Scale; the Work Motivation Scale; the Quantitative Workload and Organizational Constraints Scale; the Local and non-local Job Opportunity Scale; the Positive and Negative Affectivity Scale	Full time employment and more sprains and strains (including back injuries) result in more turnover. Hours of voluntary overtime and more than one job for pay reduce turnover. Moreover, less job satisfaction and organizational commitment led to more turnover. Finally, Magnet Recognition Award Hospitals and several other work attributes has no effect on turnover.
4	Rhéaume et al., 2011 [17]	Canada	348 new graduate nurses working in the rural province of New Brunswick	Quantitative research A cross-sectional survey	A nine-item Empowerment Scale; the Practice Environment Scale of the Nursing Work Index (PES-NWI); the Scale of Intention to Leave; a 12-item Scale Related to Place of Employment; the Socio-demographic Scale	The results showed that 49.6% of the new graduate nurses did not intend to leave their current employer, whereas 4.9% were definitely planning to leave and 45.5% expressed different levels of uncertainty. The finding indicated that new graduate nurses who identified with the goals of their organization and perceived the use of a nursing model in care were less likely to leave.
5	Unruh, & Zhang, 2013 [18]	USA	533 RNs who were newly licensed in the state of Florida in the United States	Quantitative research A correlational survey	The Personal Characteristics; the Professional Commitment Scale; the Intent to Leave Scale; the Job Difficulty Scale; the Job Demand Scale; the Job Control Scale	Job difficulty and job demand were significantly related to a greater intent to leave nursing. Nurses with positive orientation experiences and those working the day shift and more hours were less likely to intend to leave nursing.
6	Flinkman et al., 2013 [19]	Finland	3 young female registered nurses	Qualitative research A longitudinal qualitative case study	Some opened-ended questions	Nursing as a second career choice; Demanding work content and poor practice environment; The inability to identify with the stereotypical images of nurses; Life in a new career: the end of the story?
7	Ishihara et al., 2014 [20]	Japan	762 newly graduated nurses from 19 R-hospitals	Quantitative research A cross-sectional study	A questionnaire was developed by a network (International Collaboration to Study the Occupational Health of Nurses [ICOHN]) of nurse researchers from eight countries. They are the demographic scale; the nursing work index (NWI)/NWI-Revised (NWI-R); the Copenhagen psychosocial questionnaire (COPSOQ); the Copenhagen burnout inventory (CBI).	The results showed that 8.1% of respondents had a highly to extremely likely desire to leave nursing. Intention to leave was significantly associated with Nursing Work Index total, social support, work barriers, commitment to workplace, job satisfaction, and burnout.
8	Flinkman, Salanterä, 2015 [21]	Finland	15 novice registered nurses	Qualitative research An in-depth, descriptive approach	Some opened-ended questions with semi-structured	Poor nursing practice environments; lack of support, orientation and mentoring; nursing as a ‘second best’ or serendipitous career choice.
9	Numminen et al., 2016 [22]	Finland	318 newly graduated nurses	Quantitative research A descriptive, cross-sectional correlation study	The Occupational Commitment Scale (OCS); the Nurse Competence Scale (NCS); the Demographic Scale; the Turnover Intentions Scale; the Job Satisfaction Scale.	Newly graduated nurses with lower competence, disagreement of quality of care, thinking about changing profession and having other career alternatives should be recognized as a risk group for turnover. Occupational commitment has a negative significant with turnover intentions.
10	Kovner et al., 2016 [23]	USA	1335 newly licensed registered nurses	Quantitative research A panel survey design	The scale of intent to stay at job; the work motivation scale; the positive and negative affectivity scale; the job satisfaction scale; the search behavior and organizational commitment scale; the autonomy scale; the variety, workgroup cohesion, supervisory support scale; the collegial RN-MD relations, procedural justice scale; the distributive justice, promotional opportunities scale; the quantitative workload, organizational constraints scale; the local and non-local job opportunity scale.	Almost 30% of newly graduated nurses working in hospitals leave their unit during their first year of work. The five variables with the largest effects on unit retention were (1) variety (positive), (2) having another job for pay (negative), (3) first basic degree (having a bachelors or higher degree increased the probability of staying), (4) negative affectivity (positive), and (5) job satisfaction (positive).
11	Boamah, & Laschinger, 2016 [24]	Canada	215 new graduate nurses	Quantitative research A cross-sectional survey	The areas of worklife scale (AWS); the work interference with personal life (WIPL) scale; the Maslach burnout inventory-general survey (MBI-GS); the turnover intention scale.	Person-job match in six areas of worklife had a direct negative effect on burnout (emotional exhaustion and cynicism), which in turn had a direct positive effect on turnover intentions. Work-life interference also influenced turnover intentions indirectly through burnout.
12	Bontrager et al., 2016 [25]	USA	217 newly licensed registered nurses	Quantitative research A descriptive, prospective, cross-sectional survey	The demographic questionnaire; the Preceptor Role Effectiveness Scale (PRES), the Group Cohesion Scale (GCS), the Nurse Job Satisfaction Scale (NJS), and the Intent to Stay Scale	Statistically significant relationships were found among preceptor role effectiveness, job satisfaction, and intent to stay, as well as among group cohesion, job satisfaction, and intent to stay. Job satisfaction is a predictor of NLRNs’ intent to stay.
13	Fernet et al., 2017 [26]	Canada	572 French-Canadian newly registered nurses	Quantitative research A cross-sectional survey	The revised motivation at work scale (R-MAWS); Organizational and occupational commitment scale; Occupational and organizational turnover intention scale.	Autonomous and controlled motivation differently affects the intention to quit the occupation and organization at the career start. Commitment forms and targets play a complementary but relatively distinct role in the prediction of turnover intention.
14	Blegen et al., 2017 [27]	USA	1464 NLRNs employed by 97 hospitals in 3 states	Quantitative research A longitudinal study	Self-developed instruments including retention, hospital characteristics, and nurse characteristics	The overall retention rate at 1 year was 83%. Retention of NLRNs was higher in urban areas and in Magnet hospitals. The only personal characteristic that affected retention was age, with younger nurses more likely to stay.
15	Zhang et al., 2017 [28]	China	343 newly graduated nurses working in four hospitals	Quantitative research A longitudinal study	The demographic questionnaire; the intention to leave scale; the occupational stress scale; the trait coping style questionnaire; the nurse professional identity scale; the work locus of control scale	Job stress was a factor that consistently predicted intention to leave at all time points. The greater the job stress, the more likely a nurse was to harbor an intention to leave. At eight months, starting to work independently on night shifts and negative coping, these variables significantly predicted nurses’ intention to leave.
16	Hussein et al., 2019 [29]	Australia	87 new graduate nurses working in a tertiary-level teaching hospital	Quantitative research A larger pre-test and post-test study	The Manchester Clinical Supervision Scale (MCSS); the Practice Environment Scale Australia (PES-AUS); the ‘Intention to Stay in a Clinical Specialty’ survey.	Predictors of newly graduated nurses’ intention to stay in their current workplace were not having to practice beyond personal clinical capability and working in a critical care specialty. Further analysis indicated that high satisfaction with clinical supervision and high satisfaction with unit orientation were significant predictors for intention to stay.
17	Li et al., 2020 [30]	China	1313 NGRNs from 18 hospitals in six provinces	Quantitative research A cross-sectional descriptive study	Intention to leave scale; sociodemographic and professional information; 10-item proactive personality scale; the perceived person environment fit scale (PPEFS); the multidimensional scale of perceived social support (MSPSS)	6.7% of newly graduated nurses reported a high-level intention to leave. Nurses working in specialty areas (i.e., outside of medical-surgical wards) and those with a higher degree of person-organization fit showed lower intention to leave, whereas those with a higher level of education, exposure to negative workplace/life events during the previous year, and a proactive personality showed higher intention to leave.
18	Chen et al., 2021 [31]	Taiwan	331 novice nurses	Quantitative research A cross-sectional study	The demographic scale; the intention to stay scale; the nursing competence scale	The finding revealed that clinical stress, frequency of caring for patients, and taking nursing courses were correlated with novice nurses’ intention to stay in their professional careers.
19	Ulupinar, & Aydogan, 2021 [7]	Turkey	428 new graduated nurses	Quantitative research A descriptive study	Personal and professional characteristics questionnaire; the scale of adaptation to the profession; the visual analogue scale (VAS); nurses’ intention to leave scale.	The results showed that 42.5% newly graduated nurses had considered leaving nursing profession and perceive high workload, poor communication with patients and families or team members or inadequate skills and knowledge are more likely to consider turnover or leaving the profession.
20	Yu et al., 2021 [9]	Taiwan	272 newly graduated male nurses	Quantitative research A cross-sectional questionnaire survey	Participant Characteristics; Personal Resource Questionnaire (PRQ2000); Connor-Davidson Resilience Scale (CD-RISC); Professional Commitment Scale; the Scale of Intention to Stay in Nursing.	Organizational commitment has a positive influence on the intention to stay. Organizational commitment has a significant mediating effect on the relationship between social support and intention to stay. Organizational commitment has a significant mediating effect on the relationship between resilience and intention to stay.
21	Cao et al., 2021 [8]	China	361 newly graduated nurses	Quantitative research A cross-sectional study	Transition shock scale (TSS); Connor-Davidson Resilience Scale (CDRS); Perceived social support scale (PSSS); Practice environment scale of the nursing work index (PES-NWI)	Resilience, work environment, and transition shock directly and significantly affected turnover intention (direct effect). Transition shock mediated the relationships between resilience, social support, work environment, and turnover intention indirectly and significantly (indirect effect).

### Study quality

Through the literature review, no mixed method study was found. Thus, two quantitative studies and nineteen qualitative studies were included to evaluate the quality. Finally, twenty-one studies were included due to good quality. Table 2 and Table 3 provide a summary of the quality assessment.

**Table 2:** Quality assessment of quantitative studies

**Appraisal questions**	**Tominaga & Miki (2011) **[14]	**Peterson et al. (2011) **[15]	**Brewer et al. (2012)** [16]	**Rhéaume et al. (2011)** [17]	**Unruh, & Zhang (2013)** [18]	**Ishihara et al. (2014)** [20]	**Numminen et al. (2016)** [22]	**Kovner et al. (2016)** [23]	**Bontrager et al. (2016)** [25]
1. Did the study address a clearly focused question/issue?	Yes	Yes	Yes	Yes	Yes	Yes	Yes	Yes	Yes
2. Is the research method (study design) appropriate for answering the research question?	Yes	Yes	Yes	Yes	Yes	Yes	Yes	Yes	Yes
3. Is the method of selection of the subjects (employees, teams, divisions, organizations) clearly described?	Yes	Can’t tell	Yes	Yes	Yes	Yes	Yes	Yes	Yes
4. Could the way the sample was obtained introduce (selection) bias?	Yes	Yes	Yes	Yes	Yes	Yes	Yes	Yes	Yes
5. Was the sample of subjects representative with regard to the population to which the findings will be referred?	Yes	Yes	Yes	Yes	Yes	Yes	Yes	Yes	Yes
6. Was the sample size based on pre-study considerations of statistical power?	No	No	No	No	No	No	No	No	No
7. Was a satisfactory response rate achieved?	Yes	Can’t tell	Yes	Can’t tell	No	No	No	Yes	Can’t tell
8. Are the measurements (questionnaires) likely to be valid and reliable?	Yes	Yes	Yes	Yes	Yes	Yes	Yes	Can’t tell	Yes
9. Was the statistical significance assessed?	Yes	Yes	Yes	Yes	Yes	Yes	Yes	Yes	Yes
10. Are confidence intervals given for the main results?	Yes	No	Yes	No	No	No	No	No	No
11. Could there be confounding factors that haven’t been accounted for?	Yes	Yes	Can’t tell	No	Yes	Yes	Yes	Yes	Yes
12. Can the results be applied to your organization?	Yes	Yes	Yes	Yes	Yes	Yes	Yes	Yes	Yes
**Boamah, & Laschinger (2016) **[24]	**Fernet et al. (2017)** [26]	**Blegen et al. (2017)** [27]	**Zhang et al. (2017)** [28]	**Hussein et al. (2019)** [29]	**Li et al. (2020) **[30]	**Chen et al. (2021)** [31]	**Ulupinar, & Aydogan (2021)** [7]	**Yu et al. (2021)** [9]	**Cao et al. (2021)** [8]
Yes	Yes	Yes	Yes	Yes	Yes	Yes	Yes	Yes	Yes
Yes	Yes	Yes	Yes	Yes	Yes	Yes	Yes	Yes	Yes
Can’t tell	Yes	Yes	Yes	Yes	Yes	Yes	Yes	Yes	Yes
Yes	Yes	Yes	Yes	Yes	Yes	Yes	Yes	Yes	Yes
Yes	Yes	Yes	Yes	Yes	Yes	Yes	Yes	Yes	Yes
No	No	No	No	No	No	No	No	No	No
Can’t tell	Yes	Can’t tell	Yes	Yes	Yes	Yes	Yes	Yes	Can’t tell
Yes	Yes	Can’t tell	Yes	Yes	Yes	Yes	Can’t tell	Yes	Yes
Yes	Yes	Yes	Yes	Yes	Yes	Yes	Yes	Yes	Yes
No	Yes	No	Yes	Yes	Yes	No	No	No	Can’t tell
Yes	Yes	Yes	Can’t tell	Yes	Yes	Yes	Yes	Yes	Yes
Yes	Yes	Can’t tell	Yes	Yes	Yes	Yes	Yes	Yes	Yes

**Table 3:** Quality assessment of qualitative studies

**Protocol statement**	**CASP Questions**	**Paper, author (data)**	
		Flinkman et al. (2013) [19]	Flinkman, Salanterä, (2015) [21]
Adopted an appropriate method and design to meet the aims of the study	Was there a clear statement of the aims of the research?	Yes	Yes
	Is a qualitative methodology appropriate?	Yes	Yes
	Was the research design appropriate to address the aims of the research?	Yes	Yes
Used a suitable data collection strategy	Was the recruitment strategy appropriate for the aims of the research?	Yes	Yes
	Was the data collected in a way that addressed the research issue?	Yes	Yes
	Has the relationship between researcher and participants been adequately considered?	Yes	Yes
Included pertinent methods of data analysis	Was the data analysis sufficiently rigorous?	Yes	Yes
Drew conclusions and interpretations that reflected the findings of the study	Is there a clear statement of findings?	Yes	Yes
	How valuable is the research?	Valuable	Valuable
Obtained ethical approval	Have ethical issues been taken into consideration?	Yes	Yes

### Factors influencing NGRNs’ ITL nursing profession

Five categories of variables were identified from the twenty-one articles. Table 4 summarizes the relationship between these variables and NGRNs’ ITL the nursing profession.

**Table 4:** Variables related to ITL among NGRNs

**Category**	**Variables**	**Positive correlation**	**Negative correlation**	**Non-significant correlation**
Demographics	Age	Tominaga &Miki, 2011 [14] Numminen et al., 2016 [22] Fernet et al., 2017 [26]		Rhéaume et al., 2011 [17] Hussein et al., 2019 [29] Li et al., 2020 [30]
	Educational level	Li et al., 2020 [30]	Kovner et al., 2016 [23]	Tominaga &Miki, 2011 [14] Fernet et al., 2017 [26]
	Year of experience		Unruh, & Zhang, 2013 [18] Numminen et al., 2016 [22]	Fernet et al., 2017 [26]
	Professional title		Kovner et al., 2016 [23]	
	Employment status (full-time, part-time, temporary)	Rhéaume et al., 2011 [17] Brewer et al., 2012 [16]		
	Health status		Tominaga &Miki, 2011 [14] Ishihara et al., 2014 [20] Brewer et al., 2012 [16]	Unruh, & Zhang, 2013 [18]
	Shift (day/evening/night/rotating)	Zhang et al., 2017 [28]		Fernet et al., 2017 [26] Unruh, & Zhang, 2013 [18]
	Hospital (location/size)		Peterson et al., 2011 [15] Tominaga &Miki, 2011 [14]	
Support	Supervisor support		Rhéaume et al., 2011 [17] Unruh, & Zhang, 2013 [18] Ishihara et al., 2014 [20] Kovner et al., 2016 [23] Flinkman & Salanterä, 2015 [21] Li et al., 2020 [30]	Peterson et al., 2011 [15] Yu et al., 2021 [9]
	Peer support		Peterson et al., 2011 [15] Ishihara et al., 2014 [20] Flinkman & Salanterä, 2015 [21] Li et al., 2020 [30]	Yu et al., 2021 [9]
Challenges in the workplaces	Job burnout	Boamah, & Laschinger, 2016 [24]		
	Job stress	Chen et al., 2021 [31]	Zhang et al., 2017 [28]	
	Job difficulty	Unruh, & Zhang, 2013 [18] Ishihara et al., 2014 [20]		
	Job demands	Peterson et al., 2011 [15] Unruh, & Zhang, 2013 [18] Flinkman et al., 2013 [19] Ishihara et al., 2014 [20] Ulupinar, & Aydogan, 2021 [7]		
	Job competence		Numminen et al., 2016 [22] Hussein et al., 2019 [29] Chen et al., 2021 [31] Ulupinar, & Aydogan, 2021 [7]	
	Job readiness		Tominaga &Miki, 2011 [14]	
	Work group cohesion		Kovner et al., 2016 [23] Bontrager et al., 2016 [25]	
Cognitive and affective response	Organizational commitment		Brewer et al., 2012 [16] Ishihara et al., 2014 [20] Numminen et al., 2016 [22] Fernet et al., 2017 [26] Yu et al., 2021 [9]	
	Quality of care		Rhéaume et al., 2011 [17]	
	Empowerment		Rhéaume et al., 2011 [17]	
	Job satisfaction		Brewer et al., 2012 [16] Ishihara et al., 2014 [20] Kovner et al., 2016 [23] Bontrager et al., 2016 [25] Hussein et al., 2019 [29] Ulupinar, & Aydogan, 2021 [7]	
Work environment	Collegial nurse-physician relations		Rhéaume et al., 2011 [17] Ishihara et al., 2014 [20]	
	Insufficient staffing level		Rhéaume et al., 2011 [17] Flinkman et al., 2013 [19] Flinkman & Salanterä, 2015 [21]	
	Person-work environment fit		Li et al., 2020 [30]	Hussein et al., 2019 [29]
Others	Stereotypical images	Flinkman et al., 2013 [19]		
	Autonomous motivation		Flinkman et al., 2013 [19] Flinkman & Salanterä, 2015 [21] Fernet et al., 2017 [26]	
	Role model		Tominaga & Miki, 2011 [14] Bontrager et al., 2016 [25]	
	Resilience		Yu et al., 2021 [9] Cao et al., 2021 [8]	

#### Demographics

Eight demographic variables that influence NGRNs’ ITL the nursing profession are mentioned. Three studies reported that age has a positive relationship with ITL among NGRNs [14, 22, 26]. However, three other studies showed a non-significant relationship between age and ITL [17, 29, 30]. More interestingly educational level was an inconsistent influencing variable which was reported as having a positive [30] and negative [23] and non-significant correlation [14, 26] with ITL. Years of experience was an influencing factor for ITL in two studies [18, 22] but had no significant correlation in another study [26]. In addition, professional titles showed a negative relationship with ITL [23].

For employment status, the temporary employee had a higher-level ITL compared to full-time and part-time [16, 17]. Health status was another important influencing factor to ITL in three studies [14, 16, 20] while it was reported as a non-significant correlation with ITL in one study [18]. For shift (day/evening/night/rotating), the frequency nightshift of NGRNs was positively associated with intent to leave the nursing profession [28] while two other studies showed a non-significant relationship [18, 26]. The final influencing factor was the hospital characteristics, with hospital location (city center/ countryside, near/ far, etc..) and hospital size having a negative relationship to ITL.

#### Support

Seven studies reported that support (supervisor and peer) had a negative relationship with ITL [15, 17, 18, 20, 21, 23, 30]. This means that NGRNs are more likely to stay in the nursing profession if they receive more support from the head nurse and colleagues.

Peterson et al., [15] showed that peer-colleague support was a negative predictor of ITL the current job. However, supervisor support could not predict ITL in this study. It is worth noting that both supervisor and peer support were not predictors in the study by Yu et al. [9] and a significant relationship was not found in this study.

#### Challenges in the workplace

Challenges in the workplace include the negative feelings of NGRNs in clinical settings and the various demands required in the nursing profession. Job burnout [24], job stress [31], job difficulty [7, 20], and job demands [7, 15, 18–20] were all positive predictors of ITL among NGRNs. However, job stress could not predict ITL in Zhang et al.’s [28] study. In the present synthesis, the demands required of NGRNs had a negative correlation with ITL. Higher levels of job competence [7, 22, 29, 31], job readiness [14], and work group cohesion [23, 25] of NGRNs were related to lower levels of ITL.

#### Cognitive and affective response to work

As previous studies reported, both cognitive and affective have been identified as contributing to the development of intentions. Staff nurses with more emotional responses to their work prefer to stay in their current position. In our synthesis, ten studies determined that NGRNs’ cognitive and affective responses to their work were negatively related to ITL their current positions. These cognitive and affective response includes organizational commitment [9, 16, 20, 22, 26], quality of care [17], empowerment [17], and job satisfaction [7, 16, 20, 23, 25, 29].

Organizational commitment is the relative strength of an individual’s involvement in and identification with his/her organization in terms of goals and values [32]. Organizational commitment and job satisfaction were reported many times as having a significant correlation with ITL, which means that if NGRNs are satisfied with their work and committed to their organization, they will not want to leave their current job.

#### Work environment

The work environment was an important influencing factor for ITL in five studies [17, 19–21, 30] which means unfavorable perceptions of the work environment positively influence ITL. As an integral part of the work environment, disharmonious collegial nurse-physician relations, insufficient staffing levels, and personal-work environments unfit directly influence ITL. However, one study reported that there has no relationship between the work environment and ITL [29].

#### Others

In our study, we found some additional factors had an effect on ITL among NGRNs but could not be neatly grouped in the above categories. Flinkman et al. [19] found that stereotypical images were predictors of the ITL among NGRNs. The autonomous motivation was another influencing factor of NGRNs’ ITL [19, 21, 26]. While in Bontrager et al. [25] and Tominaga and Miki’s [14] study, there was a significant relationship between role models and ITL among NGRNs. And finally, resilience had a direct negative relationship with ITL [8, 9].

## Discussion

This study reviewed the influencing factor of NGRNs’ ITL nursing profession as published in research over the past decade. The findings indicated mixed and sometimes inconsistent data about NGRNs’ ITL the nursing profession. The level of ITL among NGRNs was moderate in most included studies.

Regarding the demographic characteristics of NGRNs, age and work experience were seen as important influencing factors for ITL [14, 18, 22, 26]. The findings indicated that younger NGRNs, with a lower level of work experience, are more like to leave the nursing profession than the older ones with a higher level of work experience. A possible explanation is that younger NGRNs are more exposed to repetitive assignments, have lower salaries, and participate less in decision-making [4]. These may cause them to become more dissatisfied with the profession and desire to leave their job. Conversely, older nurses may have a greater desire for stability [33], and thus express more intention to stay. Regarding educational level, the relationship between educational level and ITL among NGRNs was largely inconsistent in this review. Thus, it should be confirmed in further studies.

NGRNs employed on a temporary status, working in small hospitals, and taking more nightshifts were more likely to consider leaving their current position, however, in the theory of turnover of Price & Mueller [34] an inclusive model of turnover will result in non-significant demographic variables. Thus, demographic variables found to be the most important predictors of turnover intent or turnover should be interpreted cautiously as they may cover other meaningful factors influencing nurses’ decision-making [17].

It is worth noting that personal health status can directly lead to NGRNs leaving, especially when they have strains and sprains from their work. This finding is consistent with de Oliveira et al. [4] and Diehl et al. [35]. It is well known that good personal health status is critical to excelling well at work. Thus, it is not surprising that poor health status is associated with ITL the profession. It should be further confirmed in future research whether the health problems are caused by poor working conditions, which may prompt people to leave intentionally.

The perception of social support from supervisors and peers was negatively related to ITL. These results confirm the earlier findings of Hognestad Haaland et al. [36], Smith et al. [37], and Najafi et al. [38] that workplace support from colleagues and head nurses plays an important role for novice nurses which can help them reduce work stress and anxiety hence improving the quality of nursing care for patients and finally intent to stay in the nursing profession. Supportive leaders who consider subordinates’ feelings can reduce employee stress and increase employee job satisfaction [39, 40]. Thus, supervisor support is a crucial management strategy for reducing ITL among NGRNs. Similarly, peer support can make NGRNs adapt to the clinical setting rapidly, especially when facing challenges posed by a pandemic [41]. Moreover, the contributory factors to low retention rates of NGRNs such as burnout, stress, and job dissatisfaction may also be reduced in the presence of peer support [42].

Through this integrative review, we found that NGRNs encountered some challenges in the nursing workplace. Studies indicated that NGRNs with a higher level of job burnout, job stress, job difficulty, and job demand intended to leave their current job. Additionally, NGRNs were more vulnerable to job burnout, job stress, job difficulty, and job demand than their senior counterparts [43–46]. A possible explanation is that NGRNs don’t have enough professional experience and need to learn many clinical nursing skills in the primary phase of their career. Meanwhile, they also need more time to adapt to the clinical nursing setting. Moreover, discrepancies between what they learned at university and the reality of nursing cause difficulties, and stress and can be overwhelming for NGRNs [47]. Thus, as nursing administrators, formulating effective strategies for reducing job burnout, stress, difficulties, and job demands is necessary to keep more NGRNs in the nursing profession.

In addition, NGRNs with a higher level of job competence, job readiness, and work group cohesion were more like to stay in their current position. This finding is consistent with Chang et al. [48], Kaihlanen et al. [49], and Lyu et al. [5] who confirmed that job readiness and job competence among NGRNs are very important in facilitating a smooth transition and integration into the workplace. However, job readiness and job competence among NGRNs are not limited to clinical settings and also extend to generic skills and attributes [50]. But we need to notice that job readiness and job competence may vary according to the profession and industry. What’s more, as work group cohesion is vital for work-life, understanding and constantly striving to improve the level of work group cohesion among NGRNs is a critical assignment for nursing administrators [25].

The cognitive and affective response is presented through organizational commitment, quality of care, empowerment, and job satisfaction. The findings of this study suggest that empowerment can help minimize the job turnover intention of NGRNs. A possible explanation is that those NGRNs with higher perceived empowerment experience moral distress less often than others [51], leading to a lower turnover intention. In addition, the quality of care provided by NGRNs can enhance their confidence and lead them to accept more responsibility and accountability. This can increase job satisfaction and decrease turnover intention [3, 52]. In this integrative review, NGRNs with higher job satisfaction were less likely to leave and more committed to organizational goals. A possible explanation is that NGRNs who put in more effort to achieve organizational goals usually get more rewards. In turn, they are more satisfied with the organization and have less ITL. The reason for NGRNs not choosing to leave the nursing profession is either because they feel obligated or think that if they leave will lose too much.

In addition, a poor work environment is positively associated with NGRNs’ ITL. A supportive work environment contributes to help reduce turnover and increase employee satisfaction as well. As previous studies reported genuinely caring for them, sincerely recognizing their accomplishments, supporting them to pursue career growth, and set a clear expectation were seen as specific strategies of supportive work environment [8, 53]. Therefore, nurse administrators must create a supportive work environment for both nurse managers and peers, which can help NGRNs adapt to the clinical setting [8].

Although prior nursing scholars and clinical nurses have made many contributions and efforts to the nursing profession, the gender stereotypical is still exists. Most people think that nursing is a ‘doing’ profession and care is a ‘female’ characteristic [54]. Previous studies indicated that if such gender stereotypes increase more seriously in daily practice, nursing could perhaps be saved from nurses leaving the profession because of feeling unfulfilled [54]. Moreover, as reported by Smith et al. [37] and Lyu et al. [5] it is necessary to build a nurse role model in as many public places as possible to increase visibility. Thus, how to raise public awareness of the vital role of nurses in providing healthcare, especially in infectious disease pandemic times is a crucial issue for nursing managers. Thus, placing nurses in the public eye and setting up nurse role models should be a priority among healthcare organizations.

More interestingly, another main finding of this study is the identification of autonomous motivation as an individual factor that acts on ITL. We found that nursing was the second career choice before NGRNs enter the nursing profession. This is consistent with a previous study [55]. Most students are forced to choose a major in nursing, or they chose nursing without knowing much about the nursing profession. This means they may not be vigorously involved in their work and may be more inclined to leave it over time.

Resilience can help clinical nurses adapt and survive when suffering emergencies and stress. As a previous study reported, the more resilient nurses are, the less likely they will be to develop stress disorders [56]. Especially for NGRNs with inadequate clinical skills and knowledge, resilience acts as a buffer to protect nurses from the harmful effects of stress, which in turn decreases turnover intention.

### Limitations

The first limitation of this review was the inclusion of articles published only in English. This may have led to language bias, and some important findings published in non-English journals may have been overlooked. Also, unpublished literature was not searched. Therefore, there is a possibility of missing data.

## Conclusion

This paper reviewed twenty-one published articles reporting on ITL among NGRNs over the past decade and the associated influencing factors. The findings indicated that although the educational systems and healthcare systems have initiated efforts to retain nursing students and persuade NGRNs to stay in the nursing profession, the turnover rate is still increasing in this group. The main factors affecting NGRNs’ ITL the nursing profession include age, health status, supervisor and peer support, job demands, job competence, organizational commitment, job satisfaction, and work environment. Considering that the factors affecting NGRNs’ ITL the nursing profession are multifaceted, continued and dedicated work towards improvements can be achieved through healthcare policies, public media, training programs, clinical practice, and many other efforts. Given the current COVID-19 pandemic and more registered nurses retiring, keeping NGRNs to stay in their current positions is vital.

### Implications for nursing management

This study provides more comprehensive data for nurse administrators to develop intervention strategies to mitigate NGRNs’ turnover intention. The specific strategies include providing positive support in the clinical setting, implementing a pre-job skill training program, establishing a comfortable work environment, and improving organizational commitment and job satisfaction. Accordingly, nurse managers need to develop suitable nursing human resources development strategies, which advocate the continuing support from supervisors and peers, improve the job competence of NGRNs, create a good work environment, increase their organizational commitment.

## Data Availability

The data that support the findings of this study are available from the corresponding author upon reasonable request.

## Abbreviations

CASP: Critical Appraisal Skills Program
CEBM: Center for Evidence-Based Management
CINAHL: Cumulative Index to Nursing and Allied Health Literature
ITL: Intention to Leave
ITS: Intention to Stay
NGRNs: Newly Graduated Registered Nurses
PRISMA: Preferred reporting Items for Systematic Reviews and Meta-Analysis
RN: Registered nurses

## References

[1] Benner P (1984). From Novice to Expert: Excellence and Power in Clinical Nursing Practice.

[2] Reebals C, Wood T, Markaki A (2022). Transition to practice for new nurse graduates: Barriers and mitigating strategies. West J Nurs Res.

[3] van Rooyen DR, Jordan PJ, ten Ham-Baloyi W, Caka EM (2018). A comprehensive literature review of guidelines facilitating transition of newly graduated nurses to professional nurses. Nurse Educ Pract.

[4] Oliveira DR, Griep RH, Portela LF, Rotenberg L (2017). Intention to leave profession, psychosocial environment and self-rated health among registered nurses from large hospitals in Brazil: a cross-sectional study. BMC Health Serv Res.

[5] Lyu X, Akkadechanunt T, Soivong P, Juntasopeepun P, Chontawan R (2022). A qualitative systematic review on the lived experience of men in nursing. Nurs Open.

[6] Sandler M. Why are new graduate nurses leaving the profession in their first year of practice and how does this impact on ED nurse staffing? A rapid review of current literature and recommended reading. Can J Emerg Nurs. 2018; 41(1):23-24.

[7] Ulupinar S, Aydogan Y (2021). New graduate nurses’ satisfaction, adaptation and intention to leave in their first year: a descriptive study. J Nurs Manag.

[8] Cao X, Li J, Gong S (2021). Effects of resilience, social support, and work environment on turnover intention in newly graduated nurses: the mediating role of transition shock. J Nurs Manag.

[9] Yu H, Huang C, Chi Y, Shen Y, Chiang Y, Chang C, Lou J (2021). The mediating effects of nursing professional commitment on the relationship between social support, resilience, and intention to stay among newly graduated male nurses: a cross-sectional questionnaire survey. Int J Environ Res Public Health.

[10] Al Yahyaei A, Hewison A, Efstathiou N, Carrick-Sen D (2022). Nurses’ intention to stay in the work environment in acute healthcare: a systematic review. J Res Nurs.

[11] Whittemore R, Knafl K (2005). The integrative review: updated methodology. J Adv Nurs.

[12] Critical Appraisal Skills Program. CASP (Qualitative Review) checklist. 2019. Cited 2022 May 25. Available from: https://www.casp-uk.net/checklists.

[13] Center for Evidence-Based Management. Critical appraisal checklist for a cross-sectional study. 2014. Cited 2022 May 25. Available from: https://www.cebma.org.

[14] Tominaga MT, Miki A (2011). Factors associated with the intention to leave among newly graduated nurses in advanced-treatment hospitals in Japan. Jpn J Nurs Sci.

[15] Peterson J, McGillis Hall L, O’Brien-Pallas L, Cockerill R (2011). Job satisfaction and intentions to leave of new nurses. J Res Nurs.

[16] Brewer CS, Kovner CT, Greene W, Tukov-Shuser M, Djukic M (2012). Predictors of actual turnover in a national sample of newly licensed registered nurses employed in hospitals. J Adv Nurs.

[17] Rhéaume A, Clément L, Lebel N (2011). Understanding intention to leave amongst new graduate Canadian nurses: a repeated cross-sectional survey. Int J Nursing Stud.

[18] Unruh L, Zhang NJ (2013). The role of work environment in keeping newly licensed RNs in nursing: a questionnaire survey. Int J Nurs Stud.

[19] Flinkman M, Isopahkala-Bouret U, Salanterä S. Young registered nurses’ intention to leave the profession and professional turnover in early career: a qualitative case study. ISRN Nurs. 2013;3:1–12. 10.1155/2013/916061 PMC3762080 24027640

[20] Ishihara I, Ishibashi Y, Takahashi K, Nakashima M (2014). Effect of organizational factors and work environments on newly graduated nurses’ intention to leave. Jpn J Nurs Sci.

[21] Flinkman M, Salanterä S (2015). Early career experiences and perceptions–a qualitative exploration of the turnover of young registered nurses and intention to leave the nursing profession in Finland. J Nurs Manag.

[22] Numminen O, Leino-Kilpi H, Isoaho H, Meretoja R (2016). Newly graduated nurses’ occupational commitment and its associations with professional competence and work-related factors. J Clin Nurs.

[23] Kovner CT, Djukic M, Fatehi FK, Fletcher J, Jun J, Brewer C, Chacko T (2016). Estimating and preventing hospital internal turnover of newly licensed nurses: a panel survey. Int J Nurs Stud.

[24] Boamah SA, Laschinger H (2016). The influence of areas of work life fit and work-life interference on burnout and turnover intentions among new graduate nurses. J Nurs Manag.

[25] Bontrager S, Hart PL, Mareno N (2016). The role of preceptorship and group cohesion on newly licensed registered nurses’ satisfaction and intent to stay. J Contin Educ Nurs.

[26] Fernet C, Trépanier SG, Demers M, Austin S (2017). Motivational pathways of occupational and organizational turnover intention among newly registered nurses in Canada. Nurs Outlook.

[27] Blegen MA, Spector N, Lynn MR, Barnsteiner J, Ulrich BT (2017). Newly licensed RN retention. J Nurs Adm.

[28] Zhang Y, Wu J, Fang Z, Zhang Y, Wong FKY (2017). Newly graduated nurses’ intention to leave in their first year of practice in Shanghai: a longitudinal study. Nurs Outlook.

[29] Hussein R, Salamonson Y, Hu W, Everett B (2019). Clinical supervision and ward orientation predict new graduate nurses’ intention to work in critical care: Findings from a prospective observational study. Aust Crit Care.

[30] Li Z, Cao J, Wu X, Li F, Zhu C (2020). Intention to leave among newly graduated nurses: a descriptive, multicenter study. J Adv Nurs.

[31] Chen HM, Liu CC, Yang SY, Wang YR, Hsieh PL (2021). Factors related to care competence, workplace stress, and intention to stay among novice nurses during the coronavirus disease (COVID-19) pandemic. Int J Environ Res Public Health.

[32] Hsieh HL (2012). Building employees’ organizational commitment with LMX: the mediating role of supervisor support. Glob J Eng Educ.

[33] Engeda EH, Birhanu AM, Alene KA (2014). Intent to stay in the nursing profession and associated factors among nurses working in Amhara Regional State Referral Hospitals Ethiopia. BMC Nurs.

[34] Price JL, Mueller CW (1981). A causal model of turnover for nurses. Acad Manag J.

[35] Diehl E, Rieger S, Letzel S, Schablon A, Nienhaus A, Escobar Pinzon LC, Dietz P (2020). Health and intention to leave the profession of nursing-which individual, social and organisational resources buffer the impact of quantitative demands? A cross-sectional study. BMC Palliat Care.

[36] Hognestad Haaland G, Olsen E, Mikkelsen A (2021). The association between supervisor support and ethical dilemmas on Nurses’ intention to leave: the mediating role of the meaning of work. J Nursg Manag.

[37] Smith CM, Lane SH, Brackney DE, Horne CE (2020). Role expectations and workplace relations experienced by men in nursing: a qualitative study through an interpretive description lens. J Adv Nurs.

[38] Najafi B, Nasiri A (2023). Support experiences for novice nurses in the workplace: a qualitative analysis. SAGE Open Nurs.

[39] Bakker AB, Demerouti E (2017). Job demands–resources theory: taking stock and looking forward. J Occup Health Psychol.

[40] Basford TE, Offermann LR, Wirtz PW (2012). Considering the source: The impact of leadership level on follower motivation and intent to stay. J Leadersh Organ Stud.

[41] Varasteh S, Esmaeili M, Mazaheri M (2022). Factors affecting Iranian nurses’ intention to leave or stay in the profession during the COVID-19 pandemic. Intl Nurs Rev.

[42] Webster N, Jenkins C, Oyebode J, Bentham P, Smythe A (2019). Experiences of peer support for newly qualified nurses in a dedicated online group: study protocol. J Adv Nurs.

[43] Khalique M, Arif I, Siddiqui M, Kazmi SW (2018). Impact of workplace bullying on job performance, intention to leave, OCB and stress. Pak J Psychol Res.

[44] Lo WY, Chien LY, Hwang FM, Huang N, Chiou ST (2018). From job stress to intention to leave among hospital nurses: a structural equation modelling approach. J Adv Nurs.

[45] Minamizono S, Nomura K, Inoue Y, Hiraike H, Tsuchiya A, Okinaga H, Illing J (2019). Gender division of labor, burnout, and intention to leave work among young female nurses in Japan: a cross-sectional study. Int J Environ Res Public Health.

[46] Theodosius C, Koulouglioti C, Kersten P, Rosten C (2021). Collegial surface acting emotional labour, burnout and intention to leave in novice and pre-retirement nurses in the United Kingdom: a cross-sectional study. Nurs Open.

[47] Al-Rawajfah OM, AlBashayreh A, Al Sabei SD, Al-Maqbali M, Al Yahyaei A (2023). Role transition from education to practice and its impact on the career futures of Omani nurses. Nurse Educ Pract.

[48] Chang YC, Yeh TF, Lai IJ, Yang CC (2021). Job competency and intention to stay among nursing assistants: the mediating effects of intrinsic and extrinsic job satisfaction. Int J Environ Res Public Health.

[49] Kaihlanen AM, Elovainio M, Haavisto E, Salminen L, Sinervo T (2020). Final clinical practicum, transition experience and turnover intentions among newly graduated nurses: A cross sectional study. Nurse Educ Today.

[50] Caballero C, Walker A (2010). Work readiness in graduate recruitment and selection: A review of current assessment methods. JTLGE.

[51] Kuokkanen L, Leino-Kilpi H, Numminen O, Isoaho H, Flinkman M, Meretoja R (2016). Newly graduated nurses’ empowerment regarding professional competence and other work-related factors. BMC Nurs.

[52] Savitsky B, Shvartsur R, Findling Y, Ereli A, Hendel T (2023). Components of professional satisfaction among novice nurses. Isr J Health Policy Res.

[53] Johansen ML, Cadmus E (2016). Conflict management style, supportive work environments and the experience of work stress in emergency nurses. J Nurs Manag.

[54] van der Cingel M, Brouwer J (2021). What makes a nurse today? A debate on the nursing professional identity and its need for change. Nurs Philos.

[55] Wang H, Li X, Hu X, Chen H, Gao Y, Zhao H, Huang L (2011). Perceptions of nursing profession and learning experiences of male students in baccalaureate nursing program in Changsha China. Nurse Educ Today.

[56] Ying LY, Ramoo V, Ling LW, Nahasaram ST, Lei CP, Leong LK, Danaee M (2021). Nursing practice environment, resilience, and intention to leave among critical care nurses. Nurs Crit Care.

